# Book Review: Fluency in L2 Learning and Use (Second Language Acquisition Series, Vol. 138)

**DOI:** 10.3389/fpsyg.2021.682270

**Published:** 2021-04-21

**Authors:** Weiwei Zhang, Lawrence Jun Zhang

**Affiliations:** Faculty of Education and Social Work, University of Auckland, Auckland, New Zealand

**Keywords:** L2 fluency, fluency in an interdisciplinary approach, cognitive fluency, verbal fluency, non-verbal fluency

“For me fluency is mostly speech-related: pronunciation and the natural flow of speech.” The quote of an English as a foreign language university student from *Fluency in L2 Learning and Use* edited by Lintunen et al. (2020), exemplifies the immediate relationship between learners' perception of foreign and/or second (L2) fluency and speaking. In fact, as the editors stress, not only learners, but also many teachers and researchers intuitively and commonly link L2 fluency to speaking, although the construct is multi-faceted and should be understood across disciplinary boundaries. Bearing such an understanding in mind, the editors conceptualize the construct from various perspectives, with a clear purpose of helping readers formulate comprehensive perceptions of L2 fluency, “the central feature of L2 learning and use” (p. 2).

*Fluency in L2 Learning and Use* is the first monograph to examine L2 fluency across disciplines in the past more than two decades after the publication of *Perspective on Fluency* edited by Riggenbach (2000), one ground-breaking volume that explored L2 fluency in an interdisciplinary approach. Historically speaking, Riggenbach primarily focused on theoretical issues. Today, after L2 fluency research has embraced theoretical evolution and technical advancement, we are more welcoming of the insights into a multi-faceted understanding of state-of-the-art theories and empirical studies supported by cutting-edge methodologies for practical purposes. *Fluency in L2 Learning and Use”* coincidently delivers us such insights.

As one of us is a Professor of Applied Linguistics and TESOL (Teaching English to Speakers of Other Languages), and the other is conducting a PhD study on L2 speaking assessment, we have great interest in the topic of fluency. This is why we approached the monograph. When we searched for Riggenbach (2000) for supplementary reading via Google scholars, little information was available, and the “0 reviews” description shown on Google books further indicates its seeming undervaluation. This reminded us of a recent debate on the traditional idea of “good wine needs no bush,” and the public generally supported the necessity of promotion even for “good wine” in today' world distracted by loads of pop-up commercials. Inspired by such necessity and to avoid historical repetition, we decided to “say” something for the newly published monograph so that it can be accessed by as many readers in need like us as possible.

The monograph comprises 12 chapters, interweaving theoretical reviews with empirical studies in two strands of newness and broadness: “Current approaches to fluency,” and “interdisciplinary approach to fluency” (p. 1–2). In Chapter 1, the editors introduce L2 fluency through elaborating on Lennon's (2000) narrow/lower-order fluency vs. broad/up-order fluency and Segalowitz's (2010) cognitive fluency, perceived fluency, and utterance fluency. This reveals the theoretical framework of the monograph while equipping readers with the fundamental theory on L2 fluency. They then provide an extensive review of the construct across disciplines (Chapters 4, 6, 7, 9, 10), and demonstrate explicitly how to conduct an empirical study in a specific context (Chapters 2, 5, 8, 11).

Based on a theoretical review of L2 listening fluency, Chapter 4 offers pedagogical activities for improving L2 listening fluency and some solutions to the problems with current listening fluency assessment tasks. Chapter 6 is a reexamination of the interactional English as Lingua Franca (EFL) to conceptualize L2 interactive speaking fluency. Due to insufficient literature on non-verbal fluency, the examination of sign language fluency and factors affecting it is embedded in verbal fluency theories in Chapter 7. In Chapter 9, fluency in L2 speaking assessment is reviewed through a comparison of how the construct is interpreted in rating scales, by human raters, in L2 speaking assessment research and automatic rating systems. In Chapter 10, how fluency in L2 translational studies is defined and how it serves as a criterion in translation assessment systems is delineated. The five chapters cooperatively, while familiarizing readers with nuanced disciplinary theory on L2 fluency, illustrates how to ground L2 fluency research in a particular discipline, especially in under-researched disciplines such as EFL and sign language. Hence, the chapters are presumably the most illuminating for neophyte researchers struggling for a departure point on a L2 fluency journey. Simultaneously, the chapters are practical in guiding teachers and assessors to develop L2 listening fluency tasks, informing researchers of how to design studies on L2 interactive speaking or non-verbal fluency, helping test developers, raters and researchers interpret L2 oral fluency; and improving translators' performance with reference to an appropriate fluency criterion.

On the other hand, the four empirical studies described in the monograph substantially illustrate the actual conduct of a L2 fluency study in a particular context. Chapter 2 reports how to study learners' perceptions of fluency with a short paper-and-pencil questionnaire composed of interview-type open-ended questions to overcome time constraint. Chapter 5 exemplifies how to combine analysis of writing process via Scriptlog (a keystroke recoding tool) with that of writing product through text evaluation for investigating L2 writing fluency. It also shows how to formulate writers' profiles by integrating writing process data with individual verbalization via the stimulus recall. The two chapters are exceptionally informative to those who aim at undertaking a research project on individual's perceptions of L2 fluency and/or L2 writing fluency in terms of design issues, in particular research instruments. Chapter 8 presents a case study regarding how gestures facilitate interaction fluency through video-recoding. It complements Chapter 7, offering a window into non-verbal fluency in empirical and theoretical approaches, and thus providing readers with a quick mastery of the foundation knowledge of the under-explored discipline. Likewise, Chapter 11, which describes how singing, listening to songs and reciting song lyrics affect L2 spoken fluency in a classroom snapshot, aligning with Chapter 4, renders guidance to budding teachers in designing effective learning activities to improve their students' speaking fluency. Also, Chapter 11 speaks to Chapters 7–8, revealing the under-exploration of some disciplines in current L2 fluency research, which points to the future directions of the domain.

A mix of theory and practice, Chapter 3 displays the working mode of cognitive fluency before concretely demonstrating how to measure the internal construct through lexical access. Given the complexity of measuring cognitive fluency, information on appropriate and practical instruments is extremely laudable among those who are interested or engaged in L2 cognitive fluency studies. With a synthesis of earlier chapters, Chapter 12 informs readers of what L2 fluency essentially is in the broad and the narrow senses, and of what factors affect the construct. Such conclusive information suggests the implications of the monograph and the future directions for L2 fluency researchers and practitioners.

Overall, the editors achieve their goal of presenting a panorama of L2 fluency with state-of-the-art disciplinary theories and context-dependent cutting-edge methodologies through reviewing the literature mostly generated in the last 20 years. This concomitantly evidences the editors' claim that “this book can be reviewed as a continuation of Riggenbach's (2000) volume” (p. 2), which will facilitate the investigation of L2 fluency. Additionally, such integration of theory with methodology empowers specialists and non-specialists to disambiguate the complexity of designing and administrating a L2 fluency study. Another merit of the monograph is its reader-friendliness. The chapters are independent, and each begins with a brief introduction and ends with a concise conclusion, primarily focusing on one specific discipline. This allows readers to glean the main idea of each chapter with ease. Moreover, the monograph is well written with clarity and lucidity, intelligible for readers from a range of backgrounds and interests.

Notwithstanding its strength, one concern is the lack of focus on reading, which is in contrast with skills such as listening, writing, and speaking that are examined intensively as research contexts of L2 fluency. Except for the brief delineation in Chapter 1, reading is only mentioned occasionally elsewhere. Another concern is the structure. The three chapters (Chapters 7, 8, 11) that do not directly pertain to verbal fluency are arranged inharmoniously among chapters regarding L2 verbal fluency. Thus, there might be a need to restructure the monograph in five sections as it is presented here for an optimal organizational clarity of contents: Introduction, a theoretical approach, an empirical approach, a mixed approach, and conclusion. There are also some wording repetitions that may bother readers. For instance, “that is” is used multiple times on one page (p. 7). These issues suggest improvement in future editions. Nevertheless, in general, the monograph can be easily tailored for a broad readership in the domain of L2 fluency, including researchers, practitioners and students as an introductory reference.

## Author Contributions

WZ wrote the first draft of this book review. LZ revised it and both authors agreed to submit it.

## Conflict of Interest

The authors declare that the research was conducted in the absence of any commercial or financial relationships that could be construed as a potential conflict of interest.
